# Performance of circulating cathodic antigen (CCA) urine-dipsticks for rapid detection of intestinal schistosomiasis in schoolchildren from shoreline communities of Lake Victoria

**DOI:** 10.1186/1756-3305-3-7

**Published:** 2010-02-05

**Authors:** CJ Standley, NJS Lwambo, CN Lange, HC Kariuki, M Adriko, JR Stothard

**Affiliations:** 1 Biomedical Parasitology Division, Department of Zoology, Natural History Museum, Cromwell Road, SW7 5BD, London, UK; 2 Institute of Genetics, School of Biology, University of Nottingham, NG7 2RD, Nottingham, UK; 3 National Institute for Medical Research, Mwanza, Tanzania; 4 Invertebrate Zoology Section, Nairobi National Museum, Museum Hill, P.O. Box 40658, Nairobi, Kenya; 5 Division of Vector Borne Diseases, Ministry of Health, Nairobi, Kenya; 6 Vector Control Division, Ministry of Health, Kampala, Uganda

## Abstract

For disease surveillance and mapping within large-scale control programmes, RDTs are becoming popular. For intestinal schistosomiasis, a commercially available urine-dipstick which detects schistosome circulating cathodic antigen (CCA) in host urine is being increasingly applied, however, further validation is needed. In this study, we compared the CCA urine-dipstick test against double thick Kato-Katz faecal smears from 171 schoolchildren examined along the Tanzanian and Kenyan shorelines of Lake Victoria. Diagnostic methods were in broad agreement; the mean prevalence of intestinal schistosomiasis inferred by Kato-Katz examination was 68.6% (95% confidence intervals (CIs) = 60.7-75.7%) and 71.3% (95% CIs = 63.9-78.8%) by CCA urine-dipsticks. There were, however, difficulties in precisely 'calling' the CCA test result, particularly in discrimination of 'trace' reactions as either putative infection positive or putative infection negative, which has important bearing upon estimation of mean infection prevalence; considering 'trace' as infection positive mean prevalence was 94.2% (95% CIs = 89.5-97.2%). A positive association between increasing intensity of the CCA urine-dipstick test band and faecal egg count was observed. Assigning trace reactions as putative infection negative, overall diagnostic sensitivity (SS) of the CCA urine-dipstick was 87.7% (95% CIs = 80.6-93.0%), specificity (SP) was 68.1% (95% CIs = 54.3-80.0%), positive predictive value (PPV) was 86.1% (95% CIs = 78.8-91.7%) and negative predictive value (NPV) was 71.1% (95% CIs = 57.2-82.8%). To assist in objective defining of the CCA urine-dipstick result, we propose the use of a simple colour chart and conclude that the CCA urine-dipstick is a satisfactory alternative, or supplement, to Kato-Katz examination for rapid detection of intestinal schistosomiasis.

## Findings

Infection with *Schistosoma mansoni *is a public health concern in sub-Saharan Africa and there are still gaps in our knowledge of the occurrence of intestinal schistosomiasis at local levels. Around Lake Victoria, infection prevalence in schoolchildren can vary widely [1,2] and some areas have yet to be formally surveyed [3] so rapid assessment surveys are needed. Owing to a lack of other pragmatic alternatives, surveys have relied upon Kato-Katz preparations of single stool samples where eggs of *S. mansoni *can be visualised by microscopy [4]. While this methodology is currently recommended by WHO [5], there is now growing interest in the use of RDTs as alternative methodologies for estimating infection prevalence and intensity [6].

Only one RDT is commercially available for schistosomiasis and is manufactured by Rapid Medical Diagnostics (Pretoria, South Africa) at a cost of $ 2.3-2.8 USD per dipstick (N. van Rooyen, pers. comm.). The urine-CCA dipstick detects the presence of schistosome CCA released from adult worms excreted in the host's urine (15 μl), removing the need for faecal sampling. Formal evaluations have begun in largely field-based settings using earlier dipstick formulations [1,7-9] and as there are concerns that the high genetic variability of *S. mansoni*, potentially resulting in variation to the cathodic antigens produced, might contribute to differences in test diagnostic performance [10], it is necessary to perform evaluations in several endemic regions [6]. We have therefore examined 171 schoolchildren from eastern Lake Victoria, comparing CCA urine-dipsticks against standard Kato-Katz examination.

**Figure 1:**
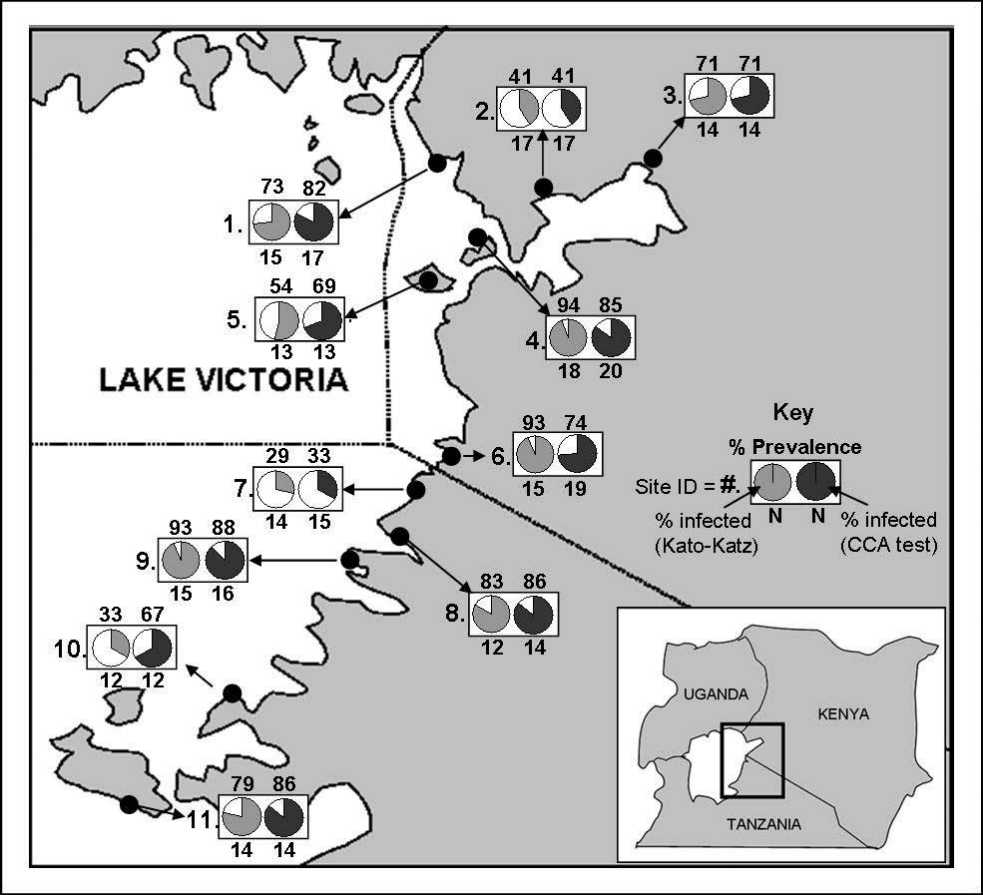
**Map of schools along the Lake Victoria shoreline visited in Kenya and Tanzania**. Map number, school name and GPS coordinates (in parentheses) are: 1. Usengi (E34.058; S0.073), 2. Asembo Bay (E34.388; S0.187), 3. Usoma (E34.718; S0.105), 4. Kolunga (E34.139; S0.428), 5. Mfangano Island (E34.015; S0.437), 6. Migori (E34.127; S1.012), 7. Mkoma (E34.030; S1.150), 8. Busanga (E33.955; S1.306), 9. Ruhu (E33.825; S1.352), 10. Majita (E33.405; S1.809) and 11. Hamuyebe (E33.064; S2.124). Pie charts indicate prevalence of schistosomiasis infection at each location as diagnosed by Kato-Katz double smear slides or urine-CCA tests; prevalence value is indicated by the number above each pie chart. The 'N' value below each pie chart indicates the surveyed sample size. The '*' indicates pooled data from two sub-sites, 5a and 5b.

During January and February of 2009, 11 shoreline schools were visited as part of a broader survey to retrieve isolates of *S. mansoni *for genotyping. Five schools were in northern Tanzania (in the Mara region) and the remaining six were in the Nyanza province of western Kenya (Fig 1).

At each school, the headmaster selected approximately 15 children for inclusion in the survey, a sample size deemed sufficient for rapid assessment protocols of schistosomiasis [3,5,11]. Surveyed children ranged in age from 6-17 years, with approximately equal boys and girls. A single stool and urine sample were requested from each child. Two thick (41.7 mg) Kato-Katz smears were prepared from each stool on the same glass slide [12], examined in the field and re-read upon return to the UK as a cross-check. Faecal egg counts were averaged across the two smears and multiplied by 24 to calculate faecal EPG. The urine sample was tested on-the-spot, without the need for transportation, and read by eye as corroborated by a second observer. Each child was offered treatment (with praziquantel, 40 mg/kg, dosed using a standard height dose pole) regardless of infection status. All participating children were provided with a single 400 mg albendazole tablet for de-worming. Ethical approval was granted by the NHS-LREC at St Mary's Hospital in London (Application 03.36), and local permits were obtained from COSTECH in Tanzania and NCST in Kenya.

Kato-Katz examination and CCA testing were in broad agreement, revealing mean prevalence of 68.6% (95% CIs = 60.7-75.7%) and 71.3% (95% CIs = 63.9-78.0%), respectively. Prevalence by school varied from 28.6% (in Mkoma) to 94.4% (in Kolunga - Number 4 on the map in Fig 1). Site 10 (Majita) displayed the widest discrepancy between diagnostic methods, with the CCA urine-dipsticks inferring a prevalence 33.4% higher than Kato-Katz examinations.

**Figure 2:**
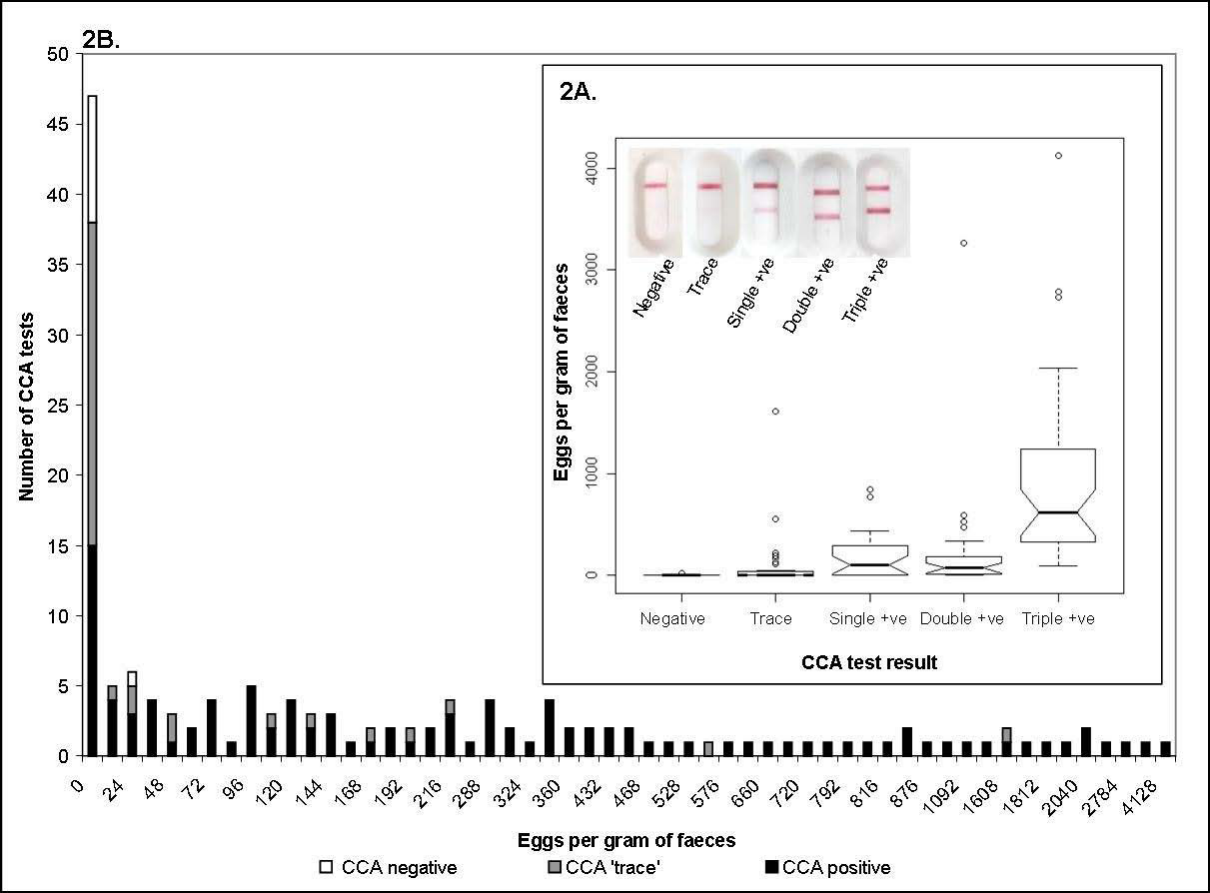
**Relationship between CCA test score and Kato-Katz faecal examination**. Figure 2a (inset): Box plot showing the relationship between CCA score and eggs per gram of faeces, for all the individuals surveyed. Representative photographs of these different test strengths can be seen in the photo montage in the top left of the Figure. Figure 2b: Stacked bar chart showing the corresponding diagnosis of the CCA tests for the children surveyed as having a particular EPG value. The CCA test only missed one positive Kato-Katz read, but 'trace' CCA results were also attributed to individuals with moderate and heavy infection intensity.

In terms of intensity of infection, the majority (50.3%) of infection intensities were 'light' (> 0 and ≤ 99 EPG), with 25.8% being 'moderate' (≥ 100 and ≤ 399 EPG) and 23.9% being 'heavy' (≥ 400 EPG). Visual recording of the intensity of the CCA test band colour that developed was more difficult, although 5.8% of schoolchildren were deemed negative, 22.8% as 'trace', 20.5% as 'single positive', 21.6% as 'double positive' and 29.2% as 'triple positive' (Fig 2a). Based on a multivariate regression model, controlling for age, sex, location and previous individual treatment history, there was a very significant positive association between faecal EPG and CCA test band colour intensity (odds ratio per 100 additional EPG = 1.06, 95% CI = 1.03-1.09, p-value = 0.0004), whereby every additional 100 eggs led to a 6% increase in the likelihood of stepping up to the next intensity category of the CCA test band.

Given the infection ambiguity of interpreting a 'trace' CCA test band, data were analysed in two permutations: 'trace' considered infection negative and 'trace' considered infection positive. The first permutation resulted in a test sensitivity (SS) of 87.7% (95% CIs = 80.6-90.3%), specificity (SP) was 68.1% (95% CIs = 54.3-80.0%), positive predictive value (PPV) was 86.1% (95% CIs = 78.8-91.7%) and negative predictive value (NPV) was 71.1% (95% CIs = 57.2-82.8%). These values are comparable to other studies [1,7,8]. However, when data were reanalysed with 'trace' as positive, mean infection prevalence was 94.2% (95% CIs = 89.5-97.2%), significantly different from both the Kato-Katz and former CCA value when 'trace' was negative, and SS became 99.1% (95% CIs = 95.6-100%), SP became 19.1% (95% CIs = 9.8-31.7%), PPV became 73.4% (95% CIs = 65.8-80.2%) and NPV became 90.0% (95% CIs = 63.2-99.4%). In Majita (Site 10), for example, considering 'trace' as positive increased local prevalence to 100%, compared the former CCA value of 67.7%, and a Kato-Katz prevalence estimate of 33.3%. Plotting EPG against the corresponding CCA test result shows that 'trace' results were assigned to faecal EPGs up to 1600. Of the schistosome egg-negative children who were CCA test positive, 53% (8/15) were considered 'single positive' while 47% (7/15) were 'double positive' (Fig 2b).

The CCA urine-dipstick is an appropriate and effective means of rapidly testing for intestinal schistosomiasis. Sensitivity and NPV were good, which is essential for not missing cases that were egg-positive although SP could be considered poorer when 'trace' was considered infection positive; this could arguably be due to the well-known insensitivity of Kato-Katz and a single stool examination missing infected cases. Increased stool sampling by obtaining three or more consecutive stools could better define 'true' infected cases [4] but such intensive sampling is not feasible for rapid assessment protocols [6]. Foremost, the convenience afforded by a single urine sample, which in itself is easier to obtain than a faecal sample, is important when conducting rapid mapping and a major advantage of the CCA urine-dipstick.

In our survey only two cases of urinary schistosomiasis were detected (all children were screened for micro-haematuria with Hemastix^®^) and each child was also co-infected with *S. mansoni*. Thus the diagnostic performance of the CCA urine-dipstick was not majorly perturbed against *S. mansoni *by the presence of *S. haematobium*, which is not easily detected by the urine CCA-dipstick in any case [8]. At the level of the individual, infection intensities were in broad alignment (see Fig 2A). Every additional 100 eggs led to a 6% increase in the likelihood of stepping up to the next intensity category of the CCA test band and to better assign CCA test band densities we propose the use of an image chart in future studies (Fig 2A).

CCA is typically released from feeding schistosome worms (inclusive of juveniles and adults) in their vomit which is later captured by the CCA urine-dipstick [9]; no doubt the production of eggs and vomit will have different daily dynamics. Male-only schistosome infections and variable proportions of egg-laying worms may interfere with a relationship between observed levels of CCA and associated faecal EPG. Interestingly, the goodness-of-fit statistic (i.e. R-squared value) for the multiple regression model only summarised *circa*. 25% of the total variance suggesting the influence of other predictive factors presently unknown. Conversely, eggs may continue to be excreted from the body when schistosome worms may no longer be extant, for example, after de-worming treatments and the summation of which eventually leads to further ambiguities. Previous research in non-human primates sets an approximate threshold in that up to 17 adult worm pairs may be present, yet Kato-Katz or CCA tests fail to detect their presence [13]. From an alternative perspective, other immune/inflammatory markers might interfere with the diagnostic SP of the CCA urine-dipstick as LewisX-trisaccharides epitopes are also picked up by the CCA urine-dipstick [14]. Thus to interpret the infection status of any individual as precisely as possible requires resources over and above that available for rapid detection assessments.

We conclude that CCA urine dipsticks should play an important role, alongside Kato-Katz examinations, in assisting in disease surveillance, and affirm that there is disease focality along the Lake Victoria shoreline. Information at the local level is important not only to support the Tanzanian National Control Programme [3] but also encourage the instigation of an equivalent intervention in Kenya.

## Abbreviations

CCA: circulating cathodic antigen; CIs: confidence intervals; COSTECH: Commission for Science and Technology; EPG: eggs per gram; NCST: National Council for Science and Technology; NPV: negative predictive value; NHS-LREC: National Health System-Local Research Ethics Committee; PPV: positive predictive value; RDTs: rapid diagnostic tests; SP: specificity; SS: sensitivity; WHO: World Health Organisation.

## Competing interests

The authors declare that they have no competing interests.

## Authors' contributions

JR and CS conceived the study, and NL and CL coordinated the fieldwork in Tanzania and Kenya, respectively. CS, MA, CNL, HCK and JRS conducted the fieldwork. MA and HK undertook initial Kato-Katz inspections which were completed by CS in the UK, who also analysed the data.

## References

[B1] Stothard JR Kabatereine NB Tukahebwa EM Kazibwe F Rollinson D Mathieson W Webster JP Fenwick A Use of circulating cathodic antigen (CCA) dipsticks for detection of intestinal and urinary schistosomiasis Acta Trop 2006 97 219 228 10.1016/j.actatropica.2005.11.004 16386231

[B2] Standley CJ Adriko M Alinaitwe M Kazibwe F Kabatereine NB Stothard JR Intestinal schistosomiasis and soil-transmitted helminthiasis in Ugandan schoolchildren: a rapid mapping assessment Geospat Health 2009 4 39 53 19908189 10.4081/gh.2009.209

[B3] Brooker S Kabatereine NB Gyapong JO Stothard JR Utzinger J Rapid mapping of schistosomiasis and other neglected tropical diseases in the context of integrated control programmes in Africa Parasitology 2009 136 1707 18 10.1017/S0031182009005940 19450373 PMC2777245

[B4] Booth M Vounatsou P N'Goran EK Tanner M Utzinger J The influence of sampling effort and the performance of the Kato-Katz technique in diagnosing *Schistosoma mansoni *and hookworm co-infections in rural Cote d'Ivoire Parasitology 2003 127 525 531 10.1017/S0031182003004128 14700188

[B5] WHO Prevention and control of schistosomiasis and soil-transmitted helminthiasis - report of a WHO expert committee WHO Technical Report Series Geneva 2002 12592987

[B6] Stothard JR Improving control of African schistosomiasis: towards effective use of rapid diagnostic tests within an appropriate disease surveillance model Trans R Soc Trop Med Hyg 2009 103 325 332 10.1016/j.trstmh.2008.12.012 19171359

[B7] Legesse M Erko B Field-based evaluation of a reagent strip test for diagnosis of *Schistosoma mansoni *by detecting circulating cathodic antigen in urine before and after chemotherapy Trans R Soc Trop Med Hyg 2007 101 668 673 10.1016/j.trstmh.2006.11.009 17368699

[B8] Stothard JR Sousa-Figueiredo JC Standley C Van Dam GJ Knopp S Utzinger J Ameri H Khamis AN Khamis IS Deelder AM Mohammed KA Rollinson D An evaluation of urine-CCA strip test and fingerprick blood SEA-ELISA for detection of urinary schistosomiasis in schoolchildren in Zanzibar Acta Trop 2009 111 64 70 10.1016/j.actatropica.2009.02.009 19426665

[B9] van Dam GJ Wichers JH Ferreira TMF Ghati D van Amerongen A Deelder AM Diagnosis of schistosomiasis by reagent strip test for detection of circulating cathodic antigen J Clin Microbiol 2004 42 5458 5461 10.1128/JCM.42.12.5458-5461.2004 15583265 PMC535219

[B10] Stothard JR Webster BL Weber T Nyakaana S Webster JP Kazibwe F Kabatereine NB Rollinson D Molecular epidemiology of *Schistosoma mansoni *in Uganda: DNA barcoding reveals substantive genetic diversity within Lake Albert and Lake Victoria populations Parasitology 2009 136 1 12 10.1017/S0031182008005167 19627628

[B11] Brooker S Kabatereine NB Myatt M Stothard JR Fenwick A Rapid assessment of *Schistosoma mansoni*: the validity, applicability and cost-effectiveness of the Lot Quality Assurance Sampling method in Uganda Trop Med Int Health 2005 10 647 658 10.1111/j.1365-3156.2005.01446.x 15960703 PMC1975759

[B12] Katz N Chaves A Pellegrino J A simple device for quantitative stool thick-smear technique in *Schistosomiasis mansoni* Rev Inst Med Trop Sao Paulo 1972 14 397 400 4675644

[B13] Wilson RA van Dam GJ Kariuki TM Farah IO Deelder AM Coulson PS The detection limits for estimates of infection intensity in schistosomiasis mansoni established by a study in non-human primates Int J Parasitol 2006 36 1241 1244 10.1016/j.ijpara.2006.07.002 16930605

[B14] Velupillai P dos Reis EA dos Reis MG Harn DA LewisX-containing oligosaccharide attenuates schistosome egg antigen-induced immune depression in human schistosomiasis Hum Immunol 2000 61 225 232 10.1016/S0198-8859(99)00136-6 10689112

